# Traumatic iridodialysis and mydriasis: Surgical reconstruction of the iris-lens-diaphragm with an iris implant and Intraocular lens

**DOI:** 10.1016/j.ajoc.2022.101545

**Published:** 2022-04-20

**Authors:** Isabella Diana Baur, Christian Steffen Mayer, Julia Storr, Ramin Khoramnia

**Affiliations:** a Department of Ophthalmology, University of Heidelberg, Im Neuenheimer Feld 400, 69120, Heidelberg, Germany; b Ophthalmology Clinic and Polyclinic, Technical University of Munich, Munich, Germany

**Keywords:** Blunt ocular trauma, Ocular contusion, Aniridia, Aphakia, Retinal detachment

## Abstract

**Purpose:**

We present the case of a severe golf related ocular injury that affected the anterior and posterior segment. Treatment included primary surgical closure of the traumatic wound and secondary reconstruction of the iris-lens-diaphragm to correct aphakia and traumatic mydriasis and iridodialysis.

**Observations:**

A 62-year-old woman presented to our clinic with severe ocular contusion after she had been hit by a golf ball in the right eye. We observed iridodialysis, traumatic mydriasis and luxation of the crystalline lens into the vitreous body as well as Berlin's edema and retinal tears. The patient underwent primary surgical closure of the traumatic wound and, 6 months later, combined Intraocular lens (IOL) and Customflex Artificial*Iris* (AI, HumanOptics, Erlangen, Germany) implantation. Uncorrected distance visual acuity was hand movement after primary surgical closure of the traumatic wound. After secondary reconstruction of the iris-lens-diaphragm, corrected distance visual acuity was 0.30 logMAR. Subjective impairment from glare could effectively be reduced and the patient was very satisfied with the aesthetic result.

**Conclusions and Importance:**

Combined AI and IOL implantation can successfully restore visual acuity and reduce sensitivity to glare while providing an excellent aesthetic result in patients with a history of severe blunt ocular trauma, even in cases with a poor visual acuity prognosis.

## 1 Introduction

Golf related ocular injuries are rather uncommon, but they often show devastating visual acuity outcomes and may lead to enucleation.^1^^,^^2^ They can be caused by golf balls and golf clubs and especially open globe injuries are associated with a poor prognosis.^3^ Blunt trauma, however, can also lead to severe visual loss, depending on the anatomical structures affected. Anterior segment trauma can lead to anterior chamber angle recession, pupillary defects, cataract or lens dislocation. Posterior segment consequences of blunt trauma include vitreous hemorrhage, retinal tears, Berlin's edema, optic nerve atrophy and macular bleeding, as well as macular holes.^4^^,^^5^

The ocular trauma score (OTS) is a diagnostic score designed to facilitate the visual acuity prognosis in the early assessment of ocular injuries.^6^ The score ranges from 1 (most severe injury) to 5 (least severe injury). It has a high prognostic accuracy^7^ and is a useful tool to counsel patients with ocular injuries.

We present a case of severe golf related blunt ocular trauma involving both the anterior and posterior segment that led to iridodialysis and traumatic mydriasis and aphakia. The patient underwent primary surgical closure of the traumatic wound and secondary reconstructive surgery to restore function and aesthetic appearance.

## 2 Case report

A 62-year-old woman presented to our clinic after she had been hit by a golf ball in the right eye. We observed a severe ocular contusion with an OTS of 2. Ultrasound revealed a dislocation of the crystalline lens into the vitreous body and vitreous hemorrhage. The injury was immediately treated surgically and intraoperatively, Berlin's edema of the peripheral retina and several retinal tears without retinal detachment as well as iridodialysis were seen. The dislocated lens was extracted and the retinal tears were treated with cryocoagulation and endolaser coagulation and the patient received an intraocular tamponade with hexafluorethane (C2F6). After primary surgical closure of the traumatic wound, the right eye was aphakic and showed traumatic mydriasis as well as iridodialysis from 3 to 6 clock hours.

**Fig. 1:**
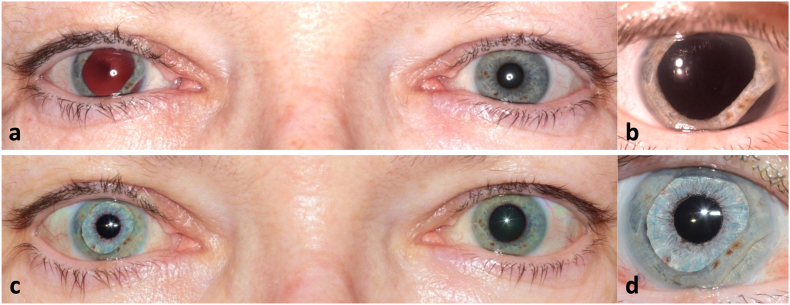
a) Binocular photography six months after primary surgical closure of the traumatic wound and before planning the pupillary and IOL reconstruction. b) Close-up of the right eye prior to secondary reconstruction of the iris-lens-diaphragm. Iridodialysis from 3 to 6 clock hours is visible c – d) Final result three months postoperatively.

Six months after the initial presentation to our clinic, the patient suffered from photophobia and decreased vision of the right eye. Fig. 1a and b show an overview and close-up photography before secondary reconstructive surgery. We found an uncorrected distance visual acuity (UDVA) of hand motion for the right eye and of 0.00 logMAR for the left eye. Corrected distance visual acuity was 0.20 logMAR with a correction of +13.0 diopters sphere for the right eye. The right eye was aphakic with traumatic mydriasis as well as iridodialysis. Funduscopy of the right eye showed an attached retina. Endothelial cell density of the right eye was 2342 cells/mm^2^ and intraocular pressure (IOP) measured with Goldmann applanation tonometry was 20 mmHg. Examination of the anterior and posterior segment of the left eye showed normal findings.

The patient was asked to rate the subjective impairment from glare and the subjective cosmetic disfigurement on a numerical rating scale from 1 to 10, with 1 standing for low and 10 standing for high severity. The patient rated the subjective impairment from glare with 9 and the subjective cosmetic disfigurement with 3.

The patient was informed about different therapeutic options including secondary IOL implantation and surgical iris reconstruction with an Artificial*Iris* (AI, HumanOptics AG, Erlangen, Germany) and the possible complications, risks and benefits. After careful consideration, the patient decided to opt for this procedure.

**Fig. 2:**
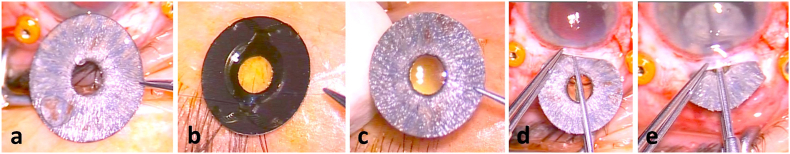
a) – c) Extraocular preparation of the AI and IOL. The AI was trephinated to the required size. The IOL was sewn to the backside of the AI and the haptics were shortened to reduce the size of the combined implants. d) – e) Insertion of both implants through a sclerocorneal tunnel at once.

There are several techniques for AI implantation that have been described in detail elsewhere.^8^ In this case, a combination of an IOL and an AI were implanted as a “sandwich”. The AI was trephinated to the required size and the IOL was sewn to the backside of the AI. The haptics of the IOL were shortened to reduce the size of the combined implant. For this technique, most commercially available IOLs designed for capsular bag implantation can be used. Fig. 2 a-c shows the extraocular preparation of the implants. Both implants were then inserted into the eye through a sclerocorneal tunnel and attached to the sclera using sutures at the three and nine o'clock position. Consequently, the stability of both implants does not depend on the IOL haptics. Fig. 2 d-e shows the insertion of the implant. We used the acrylic monofocal Aspira MC6125AS-Y IOL (HumanOptics AG, Erlangen, Germany) with an IOL power of +23,0 diopters without correction value of the results of the optic biometry in aphacic settings. The intraoperative and early postoperative course was uneventful.

Three months postoperatively, corrected distance visual acuity (CDVA) of the right eye was 0.30 logMAR, with a manifest refraction of −0.5 diopters sphere (DS) −2.0 diopters cylinder (DC) x105°. Endothelial cell density of the right eye was 1684 cells/mm^2^ and IOP was 18 mmHg. The AI was well centered (Fig. 1c and d) and the anterior segment was calm. Funduscopy and optical coherence tomography of the right eye revealed an epiretinal membrane with structural changes of the macula. The patient now rated the subjective sensitivity to glare with 5 and the subjective cosmetic disfigurement with 1 on the numerical rating scale. The patient was asked to also rate the overall satisfaction postoperatively on a numerical rating scale from 1 to 10, with 1 standing for low and 10 standing for very high satisfaction. The patient rated her overall satisfaction with 10 and stated that she would undergo the same procedure again if she had the choice. As the patient did not complain about metamorphopsia, no further surgical therapy was planned for treatment of the epiretinal membrane at this time.

## 3 Discussion

Considering the severity of the trauma that the patient had suffered, we observed a very good postoperative outcome. With an OTS of 2 at the initial presentation, the estimated probability of achieving this visual acuity level was only 15%.^6^

Iridodialysis is usually treated using sutures and can be combined with pupilloplasty to treat traumatic mydriasis. A variety of suturing techniques have been proposed.^9^, ^10^, ^11^, ^12^, ^13^ In some cases, however, surgical iris reconstruction using sutures can be particularly challenging. Suturing techniques require enough iris tissue to allow for adequate reconstruction limiting postoperative glare and providing an aesthetically pleasing outcome with a well centered pupil. In this case with rather extensive iridodialysis as well as pronounced traumatic mydriasis and aphakia we were faced with a rather complex situation. The use of sutures alone was therefore not considered a suitable option, because this would not have treated the aphakia. We decided to use a different approach to correct both iridodialysis and traumatic mydriasis as well as aphakia in one step.

Secondary IOL implantation and surgical iris reconstruction with an AI implant successfully restored visual acuity and reduced our patient's sensitivity to glare, while also having yielded an excellent aesthetic result. Our observations are in good agreement with previously published results after AI implantation. The AI implant can effectively reduce sensitivity to glare and improve contrast sensitivity.^14^^,^^15^ The cosmetic results are excellent, as the implant is customized to match the remaining iris tissue or the fellow eye.^14^^,^^16^^,^^17^ A significant increase in visual acuity after combined AI and IOL implantation has been reported,^15^ which can mainly be attributed to the correction of aphakia or cataract, as the pupil diameter of 3.35 mm is too large to create a pinhole effect.^18^

In cases like this with suture fixated AI, the use of an AI with a polymer fiber meshwork is recommended. The fiber meshwork prevents cutting through of the sutures.^8^ It has been previously shown that the suturing of an IOL to the AI does not impair the optical quality.^19^ Combined implantation of an IOL and Artificial Iris provides predictable postoperative refraction without the need for a correction factor.^20^

A range of complications have been reported after AI implantation including decentration of the implant, macular edema, decreased visual acuity, worsening or onset of glaucoma, corneal decompensation and retinal detachment.^21^ A progressive enlargement of the original pupillary aperture, the so-called residual iris retraction syndrome (RITS) has been observed as a long-term complication.^22^ The risk of complications may vary between patients depending on the concomitant injuries. Glaucoma patients for example are at a higher risk for postoperative IOP elevation following cataract surgery or vitrectomy.^23^, ^24^, ^25^ The same applies to the combined IOL and AI implantation. Patients should be educated about possible risks and benefits before surgery. In the case we are reporting, secondary iris reconstruction and IOL implantation was performed several months after the initial trauma, when a stable situation had been achieved.

Alternative surgical therapeutic options include the iris prosthesis from Ophtec (Groningen, the Netherlands). The implant is available with an optic correction in a wide diopter range and allows to correct aniridia und aphakia in one step. There are different colors and patterns available, but in contrast to the implant we used, the iris prothesis cannot be customized to achieve an optimal aesthetic result. The different aniridia implants from Morcher (Stuttgart, Germany) can correct aphakia and aniridia, but they are only available in black color. Those implants require a larger incision size as they are made of a rather rigid material.

We decided to use the Customflex ArtificialIris from HumanOptics, as it allows an individualized surgical approach and provides excellent aesthetic outcomes.

## 4 Conclusions

The combined implantation of an AI and IOL in a case of traumatic mydriasis and iridodialysis due to a golf related severe blunt trauma could effectively reduce glare sensitivity and improve visual acuity. The patient was very satisfied with the functional and aesthetic outcome. This case demonstrates that surgical iris reconstruction and aphakia correction could be performed successfully even in a patient with a history of severe ocular trauma and reduced visual acuity prognosis.

## Patient consent

Patient consent to publish an account of this case was not obtained as our report does not contain any information that could lead to the identification of the patient. Retrospective review of this case was done in accordance with the Declaration of Helsinki.

## Funding

The Department of Ophthalmology of the University of Heidelberg was supported by the 10.13039/501100007316Klaus Tschira Foundation. The publication fee was covered by HumanOptics.

## Authorship

All authors attest that they meet the current ICMJE criteria for authorship.

## Intellectual property

We confirm that we have given due consideration to the protection of intellectual property associated with this work and that there are no impediments to publication, including the timing of publication, with respect to intellectual property. In so doing we confirm that we have followed the regulations of our institutions concerning intellectual property.

## Research ethics

We further confirm that any aspect of the work covered in this manuscript that has involved human patients has been conducted with the ethical approval of all relevant bodies and that such approvals are acknowledged within the manuscript.

## Declaration of competing interest

CM reports travel grants and lecture fees from HumanOptics. IDB, JS and RK declare that they have no competing interests.

## References

[1] Jayasundera T., Vote B., Joondeph B. (2003). Golf‐related ocular injuries. Clin Exp Ophthalmol.

[2] Crane E.S., Kolomeyer A.M., Kim E., Chu D.S. (2016). Comprehensive review of golf-related ocular injuries. Retina.

[3] Mieler W.F., Nanda S.K., Wolf M.D., Harman J. (1995). Golf-related ocular injuries. Arch Ophthalmol.

[4] Atmaca L., Yilmaz M. (1993). Changes in the fundus caused by blunt ocular trauma. Ann Ophthalmol.

[5] Khoramnia R., Mohrenfels C.W., Salgado J.P., Lanzl I.M., Lohmann C.P., Mayer C. (2010). [Spontaneous closure of traumatic macular holes. Two cases]. Ophthalmologe.

[6] Scott R. (2015). The ocular trauma score. Community Eye Health.

[7] Man C.Y.W., Steel D. (2010). Visual outcome after open globe injury: a comparison of two prognostic models—the Ocular Trauma Score and the Classification and Regression Tree. Eye.

[8] Mayer C., Tandogan T., Hoffmann A.E., Khoramnia R. (2017). Artificial iris implantation in various iris defects and lens conditions. J Cataract Refract Surg.

[9] Ravi KV. Sewing Machine Technique for Iridodialysis Repair.

[10] Zeiter J.H., Shin D.H., Shi D.X. (1993).

[11] Narang P., Agarwal A., Agarwal A., Agarwal A. (2018). Twofold technique of nonappositional repair with single-pass four-throw pupilloplasty for iridodialysis. J Cataract Refract Surg.

[12] Frisina R., Parrozzani R., Tozzi L., Pilotto E., Midena E. (2020). Pupil cerclage technique for treatment of traumatic mydriasis. Eur J Ophthalmol.

[13] Lumi X., Lumi A., Pajic S.P. (2021). Iris cerclage pupilloplasty and IOL implantation for traumatic mydriasis and aphakia after the blunt trauma of the eye. Indian J Ophthalmol.

[14] Mayer C.S., Reznicek L., Hoffmann A.E. (2016). Pupillary reconstruction and outcome after artificial iris implantation. Ophthalmology.

[15] Mayer C.S., Hoffmann A.M., Prahs P., Reznicek L., Khoramnia R. (2020). Functional outcomes after combined iris and intraocular lens implantation in various iris and lens defects. BMC Ophthalmol.

[16] Yildirim T.M., Khoramnia R., Masyk M., Son H.-S., Auffarth G.U., Mayer C.S. (2020). Aesthetics of iris reconstruction with a custom-made artificial iris prosthesis. PLoS One.

[17] Baur I.D., Mayer C.S., Storr J., Khoramnia R. (2021). [Artificial Iris implantation in a patient with Iatrogenic Iris defect following cataract surgery]. Klin Monbl Augenheilkd.

[18] Miller D., Johnson R. (1977). Quantification of the pinhole effect. Surv Ophthalmol.

[19] Mayer C., Son H.-S., Labuz G., Yildirim T.M., Auffarth G.U., Khoramnia R. (2020). In vitro optical quality assessment of a monofocal IOL sutured to an artificial iris. J Cataract Refract Surg.

[20] Mayer C., Khoramnia R. (2021). Double prosthesis implantation”: biometry and refractive outcomes in combined intraocular lens and artificial Iris surgery. Clin Ophthalmol.

[21] Mayer C.S., Laubichler A.E., Khoramnia R. (2018). Challenges and complication management in novel artificial Iris implantation. J Ophthalmol.

[22] Mayer C.S., Laubichler A.E., Masyk M., Prahs P., Zapp D., Khoramnia R. (2019). Residual Iris retraction syndrome after artificial Iris implantation. Am J Ophthalmol.

[23] Annam K., Chen A.J., Lee I.M., Paul A.A., Rivera J.J., Greenberg P.B. (2018). Risk factors for early intraocular pressure elevation after cataract surgery in a cohort of United States Veterans. Mil Med.

[24] Grzybowski A., Kanclerz P. (2019). Early postoperative intraocular pressure elevation following cataract surgery. Curr Opin Ophthalmol.

[25] Jabbour E., Azar G., Antoun J., Kourie H.R., Abdelmassih Y., Jalkh A. (2018). Incidence and risk factors of ocular hypertension following pars plana Vitrectomy and silicone oil Injection. Ophthalmologica.

